# Endothelial progenitor cells (EPC) in sepsis with acute renal dysfunction (ARD)

**DOI:** 10.1186/cc10100

**Published:** 2011-03-11

**Authors:** Susann A Patschan, Daniel Patschan, Johanna Temme, Peter Korsten, Johannes T Wessels, Michael Koziolek, Elvira Henze, Gerhard A Müller

**Affiliations:** 1 Department of Nephrology and Rheumatology, University Medical Center Göttingen, Robert-Koch-Straße 40, 37075 Göttingen, Germany; 2 Core Facility 'Molecular & Optical Live Cell Imaging (MOLCI)', University Medical Center Göttingen, Robert-Koch-Straße 40, 37075 Göttingen, Germany

## Abstract

**Introduction:**

Sepsis is characterized by systemic microvascular dysfunction. Endothelial progenitor cells (EPCs) are critically involved in maintaining vascular homeostasis under both physiological and pathological conditions. The aim of the present study was to analyze the endothelial progenitor cell system in patients suffering from sepsis with acute renal dysfunction.

**Methods:**

Patients with newly diagnosed sepsis were recruited from the ICU in a nonrandomized prospective manner. Blood samples were obtained within the first 12 hours after the diagnosis of sepsis. For quantifying endothelial progenitor cells (EPCs), CD133^+^/Flk-1^+ ^cells were enumerated by cytometric analysis. Analysis of EPC proliferation was performed by a colony-forming units (CFU) assay. Blood concentrations of proangiogenic mediators were measured by ELISA. Acute renal dysfunction was diagnosed according to the Acute Kidney Injury Network (AKIN) criteria. Depending on the overall mean creatinine concentration during the stay at the ICU, patients were either assigned to a 'normal creatinine group' or to a 'high creatinine group'. Survival rates, frequency of dialysis, the simplified acute physiology score (SAPS) II scores, and different laboratory parameters were collected/used for further clinical characterization

**Results:**

Circulating EPCs were significantly higher in all sepsis patients included in the study as opposed to healthy controls. Patients within the 'high creatinine group' showed an even more pronounced EPC increase. In contrast, EPC proliferation was severely affected in sepsis. Neither total circulating EPCs nor EPC proliferation differed between patients requiring dialysis and patients without renal replacement therapy. Cell numbers and cell proliferation also did not differ between surviving patients and patients with sepsis-related death. Serum levels of vascular endothelial growth factor (VEGF), stromal derived factor-1 (SDF-1), and Angiopoietin-2 were higher in sepsis than in healthy controls. Sepsis patients within the 'high creatinine group' showed significantly higher mean serum levels of uric acid.

**Conclusions:**

Sepsis significantly affects the endothelial progenitor cell system, as reflected by increased EPC numbers, increased concentrations of proangiogenic mediators, and reduced proliferative capacity of the cells. This occurs independently from the frequency of dialysis and from patient survival. Increased serum levels of uric acid are possibly responsible for stronger EPC mobilization in sepsis patients with higher average creatinine levels.

## Introduction

Sepsis, defined as systemic inflammatory response syndrome of infectious origin [1], is characterized by systemic microvascular dysfunction [2,3]. Possible consequences involve reduced microvascular blood flow, thrombocyte aggregation, and activation of coagulation [4,5]. Finally, severe organ failure can occur [6].

Endothelial progenitor cells (EPCs), although heterogenous in phenotypical and biological properties [7-10], are critically involved in maintaining vascular homeostasis and in mediating macro- and microvascular repair under both physiological and pathological conditions [11-14]. This has been documented in numerous experimental and clinical studies over the past 10 years [11,12,15,16]: impaired endothelial progenitor cell proliferation has been shown in patients with macrovascular damage such as coronary artery and cerebrovascular disease [15,17]. Patients with chronic renal failure, which are at higher risk for artherosclerosis than healthy individuals, display lower proliferation of blood derived EPCs [18]. In acute ischemic renal failure, which is characterized by postischemic hypoperfusion of peritubular capillaries, renal function could be preserved by systemic administration of both mature endothelial cells and endothelial progenitor cells [16,19]. EPCs have also been documented to be involved in glomerular endothelial repair: bone marrow transplantation experiments in animals suffering from experimental glomerulonephritis ('Thy-1 glomerulonephritis') revealed that relevant numbers of damaged glomerular endothelial cells are replaced by bone marrow-derived cells [20,21]. In addition, EPCs have been proven to actively mediate endothelial regeneration in a model of thrombotic microangiopathy [22]. Finally, the cells have been documented to mediate repair of damaged renal tissue in acute ischemic renal failure [16,23,24]. It could be shown that tubular epithelial damage can be prevented by systemic administration of EPCs in such a situation [24].

Two newer studies reported increased peripheral endothelial progenitor cells in patients suffering from sepsis [25,26]. Cell numbers correlated with survival [26] and severity of the disease [25]. Nevertheless, the authors did not particularly analyze the possible impact of sepsis-associated acute renal dysfunction on EPC proliferation and total numbers of circulating EPCs. Therefore, the aim of the present study was to analyze the endothelial progenitor cell system in patients suffering from sepsis with acute impairment of renal function.

## Materials and methods

### Patients and blood samples

Blood samples were obtained from 40 patients with sepsis in a nonrandomized prospective manner. Sepsis was defined as systemic inflammatory response syndrome (SIRS) of infectious origin [1]. Therefore, beside fulfilling the criteria of SIRS [6], all patients showed at least one positive blood culture for either Gram-positive or Gram-negative bacteria. Patients with pre-existing ESRD (end stage renal disease) were not included in the study. This was of particular importance since previous studies showed reduced EPC proliferation in uremic patients [18]. All patients were recruited at the intensive care unit over a period of 15 months. The study protocol was approved after review by the local ethics committee. The investigation conformed to the principles outlined in the Declaration of Helsinki and written informed consent was obtained from each subject. Healthy, age- and gender-matched individuals served as controls. For the studies, each patient (and the respective controls) provided four blood samples (7.5 ml each), from which two (2 × 7.5 ml) were used for endothelial and myelomonocytic cell studies, and two (2 × 7.5 ml) were used for performing routine laboratory (see biochemical and hematological tests) as well as immunological studies. For quantifying renal function, urine was collected over a period of 24 hours and creatinine clearance was calculated according to the formula by Cockcroft-Gault [27]. The severity of acute renal damage, if present, was evaluated using the AKIN (Acute Kidney Injury Network) criteria. All blood samples were drawn within 12 hours after the diagnosis of sepsis. For further clinical characterization different parameters, such as C-reactive protein and the SAPS (Simplified Acute Physiology Score) II scores, were documented at the time blood was drawn. In addition, the SAPS II scores were documented in all patients on a daily basis. In all patients sepsis-related death was documented as an outcome parameter. Indications for dialysis were the presence of one or more of the following criteria: refractory hyperkalemia, increases of serum creatinine >3 mg/dl and/or of blood urea nitrogen >100 mg/dl at any given time point, and signs/symptoms of fluid overload due to diminished urine output, respectively.

### Flow cytometry

**Figure 1:**
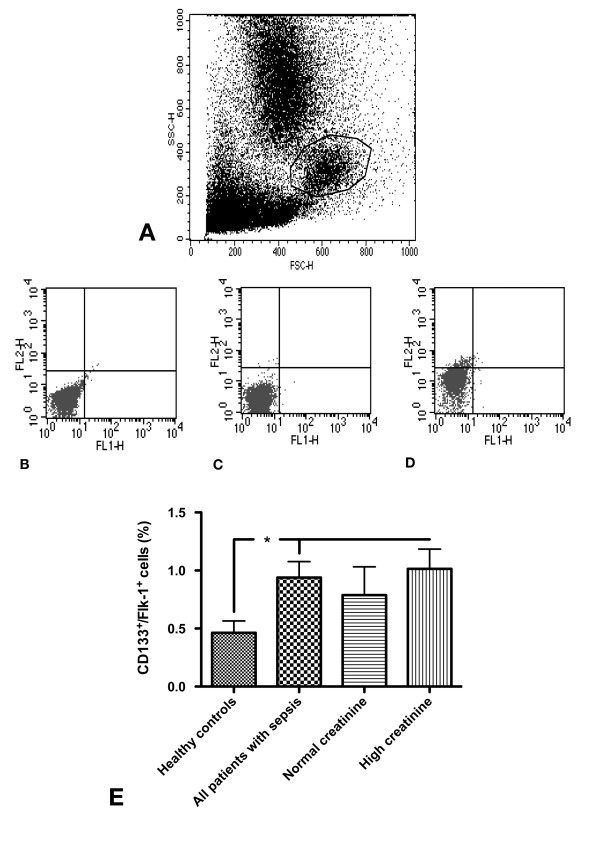
**CD133^+^/Flk-1^+ ^cells (circulating EPCs) in patients with sepsis as compared to healthy controls**. For quantification of peripheral circulating EPCs, all myelomonocytic cells were gated (A). Gated cells were analyzed without antibody staining (B), using the isotype control (C), and with Flk-1 FITC and CD133 (+secondary antibody) combined. Circulating EPCs in all patients suffering from sepsis and in sepsis patients within the 'high creatinine group' were significantly higher than in healthy controls. There was no statistically significant difference in EPCs between healthy controls and patients within the 'normal creatinine group'. (Results as mean ± SEM).

For performing flow cytometry, mononuclear cells (MNCs) were isolated by density gradient centrifugation using Histopaque-1077 solution (Sigma Diagnostics, St. Louis, MO, USA) from approximately 7.5 ml of heparinized peripheral blood. Cells were primarily incubated for one hour on ice with one or more of the following antibodies: rabbit anti CD133 (ab16518 - Abcam, Cambridge, UK), mouse anti-human VEGFR2 (FAB 3571F - R&D Systems, Minneapolis, MN, USA), followed by secondary incubation with PE-conjugated goat anti-rabbit Fab (VEGFR, 111-116-144 - Jackson ImmunoResearch Laboratories, Inc., West Grove, PA, USA) for 30 minutes on ice, respectively. After incubation, cells were washed with PBS-BSA 1% (w/v). Data were acquired using a FACScalibur cytometer (Becton Dickinson, Heidelberg, Germany) equipped with a 488 nm argon laser and a 635 nm red diode laser and analyzed using CellQuest software (Becton Dickinson, San Jose, CA, USA). The setup of FACScalibur was performed according to the manufacturer's instructions using unstained and single-antibody stained cells. Specificity of staining was controlled by incubation with isotype-matched immunoglobulins. To quantify total peripheral endothelial cells, the numbers of Flk-1 positive cells, to quantify EPCs, the numbers of CD133/Flk-1 double-positive cells within the myelomonocytic cell population were counted [28]. For this purpose, unstained mononuclear cells were first gated for the myelomonocytic subpopulation. With regard to the literature, EPCs (in our study: so-called 'early outgrowth' EPCs [9]) are not substantially detectable within the lymphocytic subpopulation [29]. The gating strategy was adapted to the ISHAGE guidelines for the enumeration of CD34+ cells [30]. Next, single-antibody stained cells were gated as well in order to recognize possible unspecific fluorescence signals and in order to define a threshold between positive and negative signals. Finally, cells incubated with anti-CD133 and anti-Flk-1 were measured and in each analysis at least 1.5 × 10^6 ^cells were counted. The methodological procedure is summarized in Figure 1.

### Analysis of EPC proliferation (colony-forming units (CFU) assay)

The assay was performed by using the EndoCult Liquid Medium Kit^® ^(StemCell Technologies, Vancouver, BC, Canada) using the manufacturer's protocol. MNCs were resuspended in complete EndoCult medium and seeded at 5 × 10^6 ^cells/well on fibronectin-coated tissue culture plates (BD Biosciences, Rockville, MD, USA). After 48 hours, wells were washed with media and nonadherent cells were co-llected. Nonadherent cells were plated in their existing media at 10^6 ^cells/well in 24-well fibronectin-coated tissue culture plates for three days. Only colonies with at least 20 cells, containing rounded cells in the middle and elongated cells at the periphery, were considered as CFU-EC colonies. The numbers of colonies a-ppearing after this period were counted [28]. At least two members of the laboratory staff evaluated the numbers of CFU-ECs. They were blinded for the diagnosis and status of the investigated patients/controls.

In all patients, the phenotype of cells within the colonies was determined in more detail. For this purpose, cells were characterized by the uptake of DiI-labeled acetylated low density lipoprotein (acLDL) (Invitrogen, Carlsbad, CA, USA) and binding of FITC-labeled UE lectin (Sigma Diagnostics, St. Louis, MO, USA). Cells were first incubated with 10 μg/ml DiI-ac-LDL at 37°C for 1 hour and later fixed with 2% formaldehyde for 10 minutes, followed by incubation with UE lectin at 37°C for 1 hour. The number of Dil-acLDL^+^/UE lectin^+ ^cells was counted by laser scanning microscopy using an inverted fluorescence microscope IX-71 (Olympus Deutschland GmbH, Hamburg, Germany) equipped with the appropriate excitation and emission filters (AHF Analysentechnik, Tuebingen, Germany).

### Enzyme-linked immunosorbent assay (ELISA)

Commercial ELISA tests were purchased for the assessment of vascular endothelial growth factor (VEGF), stromal-derived factor-1 (SDF-1), fibroblast growth factor (FGF) (all from USCN, Wuhan, China), and Angiopoietin-1 and -2 (Alpco, Salem, NH, USA) serum levels. ELISA tests were performed according to the manufacturer's protocol.

### Biochemical and hematological tests

Biochemical and hematological tests were performed in the Central Laboratories of the University Hospital Göttingen, according to the institutional guidelines.

### Statistical analysis

All values are expressed as mean ± SEM. The means of two populations were compared by the Mann-Whitney U-Test. In order to compare outcome variables, Fisher's test was performed. Correlation analysis was performed by Spearman's correlation analysis. Differences between the two groups were considered significant at *P *< 0.05, positive correlation was considered at r = 1.

## Results

### Patients characteristics

A total of 40 patients with sepsis (17 female, 23 male, mean age 69 ± 1.9 years) was included in the study. All patients were recruited from the intensive care unit. Out of these 40 patients, 25 patients developed acute renal failure during the course of the disease. In all patients serum creatinine was measured on a daily basis. Depending on the overall mean creatinine concentration 12 patients were assigned to the 'normal creatinine group' (creatinine ≤1 mg/dl), the mean serum creatinine was 0.7 ± 0.05 mg/dl. Twenty-eight patients were assigned to the 'high creatinine group' (creatinine >1 mg/dl), the mean serum creatinine was 2.5 ± 0.28 mg/dl. Within the 'high creatinine group', 15 patients were male (mean age 72 ± 3.8) and 13 were female (mean age 69 ± 3.6). The mean AKIN score was significantly higher in the 'high creatinine group' as opposed to the 'normal creatinine group' (2.94 ± 0.28 vs. 2.0 ± 0.06, *P *= 0.02). The frequency of dialysis was 6/12 patients (50%) in the 'normal creatinine group' and 19/28 (67.8%) in the 'high creatinine group'. Dialysis frequency did not significantly differ between the two groups. Survival analysis revealed that mortality rates as well did not differ between patients within the 'normal creatinine group' and patients within the 'high creatinine group'. There were also no differences in survival between patients requiring dialysis and patients without the need for dialysis. Patients within the 'high creatinine group' showed significantly higher mean serum levels of uric acid (9.1 ± 2.9 mg/dl vs. 4.5 ± 1.5 mg/dl, *P *< 0.0001). Patients' baseline characteristics are summarized in Table 1.

**Table 1:** Patients' characteristics

Patient	Mean CRP (mg/dl)	SAPS II score	Mean serum creatinine (mg/dl)	Serum uric acid (mg/dl)	Dialysis	death
1	187	33	0.36	1.2	+	-
2	108	34	0.54	na	-	-
3	168	32	0.6	4.6	-	+
4	116	34	0.6	5.5	+	+
5	217	44	0.6	4.5	+	+
6	203	31	0.65	6.8	-	+
7	319	45	0.69	5.3	+	+
8	226	21	0.8	3.7	-	-
9	209	45	0.8	3.3	+	+
10	179	29	0.9	na	-	-
11	150	49	0.9	5.6	+	+
12	248	27	1	4.1	-	-

13	380	28	1.07	6	-	-
14	66	48	1.1	7.1	+	+
15	193	65	1.11	7.6	+	+

16	186	24	1.25	10.3	+	-
17	186	37	1.28	6.7	-	-

18	173	25	1.32	11.9	-	-
19	166	25	1.4	na	-	-

20	129	29	1.4	na	-	-
21	519	23	1.41	5.7	-	-
22	199	48	1.43	4.4	+	-
23	22	36	1.64	11.5	+	-
24	127	45	1.7	7	+	-
25	104	24	1.9	na	-	-
26	235	35	2.23	13.9	+	+
27	92	42	2.3	na	+	-
28	75	43	2.5	na	+	+
29	411	50	2.77	7.2	+	-
30	184	28	2.8	16.3	+	-

31	419	24	2.97	8.2	-	-
32	309	68	3	7.6	+	+

33	169	52	3.2	na	+	+
34	328	34	3.41	8	+	-

35	297	54	3.55	13.3	+	-
36	110	25	3.63	11.3	+	-
37	294	50	4.11	8.2	+	+
38	31	45	4.71	9	-	+
39	25	29	6.47	11	+	-
40	70	24	6.68	7.4	+	-

Patients' characteristics: 12 patients were assigned to the 'normal creatinine group', whereas 28 were assigned to the 'high creatinine group'. There were no differences in age, SAPS II score, CRP, frequency of dialysis, or survival between the two groups. Patients within the 'high creatinine group' showed significantly higher mean serum levels of uric acid (CRP, C-reactive protein; SAPS II, simplified acute physiology score II; Data as mean ± SEM; na, not available).

### Circulating endothelial progenitor cells (EPCs)

For quantifying circulating EPCs, we measured CD133^+^/Flk-1^+ ^myelomonycytic cells. Since CD133 [31], as compared to CD34, has not been shown to be expressed by mature endothelial cells, we decided to discard CD34 as a marker of EPCs in our analyses [28]. In a recently published manuscript on EPCs in hypertensive patients with microalbuminuria [32], the authors measured CD34^+^/CD133^+ ^cells. Although such cells also give rise to EPCs during further stages of development, they represent precursors of monocytes as well. In this regard, enumeration of CD34^+^/CD133^+ ^cells does not exclusively represent the endothelial lineage. For that reason, these cells were not quantified in our current study. The percentages of total circulating endothelial progenitor cells (CD133^+^/Flk-1^+ ^cells in the percentage of all myelomonocytic cells) in all patients suffering from sepsis and in sepsis patients within the 'high creatinine group' were significantly higher than in healthy controls (0.93 ± 0.13% vs. 0.46 ± 0.1%, *P *= 0.02 (% of total MNC) and 1.0 ± 0.1% vs. 0.46 ± 0.1%, *P *= 0.01 (% of total MN)]). There was no statistically significant difference in EPCs between healthy controls and patients within the 'normal creatinine group' (Figure 1).

Further analysis revealed that there were no differences in total peripheral circulating EPCs between patients requiring dialysis as compared to those without the need for renal replacement therapy (data not shown). There were also no differences in circulating EPCs between patients that had died from sepsis as compared to patients who had not (data not shown).

### Proliferative activity of circulating EPCs (number of CFU-ECs)

**Figure 2:**
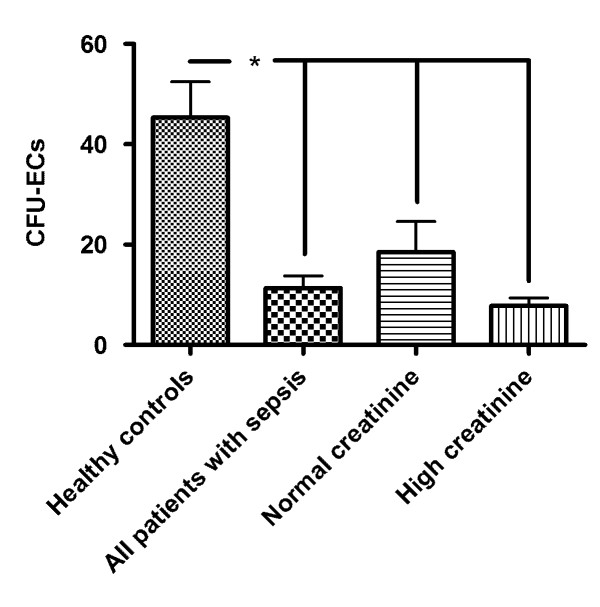
**Proliferative activity of peripheral circulating EPCs in sepsis patients as compared to healthy controls**. CFU-ECs (colony-forming unit endothelial cells) were lower in sepsis patients than in healthy controls. The differences appeared independently from the mean serum creatinine levels, subgroup analysis revealed that (I) all patients with sepsis, and (II) patients within the 'normal creatinine group', and (III) patients within the 'high creatinine group' showed significant impairment of endothelial progenitor cell proliferation as compared to healthy controls. Patients within the 'high creatinine group' showed an even more pronounced reduction in EPC proliferation than patients within the 'normal creatinine group' (Results as mean ± SEM).

Our previous studies [28] and studies performed by others [18] had shown that circumstances characterized by macro- and microvascular damage are associated with impaired endothelial progenitor cell proliferation. In sepsis, both the function and structure of small blood vessels within the whole organism can severely be affected [1,2]. Therefore, in order to assess the proliferative potential of the endothelial progenitor cell system in our sepsis patients, a colony-forming unit (CFU) assay was performed [33]. The so-called CFU assay is a widely accepted method to evaluate proliferation of 'early outgrowth' EPCs (which were analyzed in our series of experiments). This has been documented in numerous previous studies [8-10,13,14,28,33]. The analysis clearly showed lower numbers of CFU-ECs (colony-forming unit endothelial cells) in patients with sepsis than in healthy controls. The differences appeared independently from the mean serum creatinine levels, subgroup analysis revealed that (I) all patients with sepsis, (II) patients within the 'normal creatinine group', and (III) patients within the 'high creatinine group' showed significant impairment of endothelial progenitor cell proliferation as compared to healthy controls (11.3 ± 2.3, and 18.5 ± 6.1, and 7.8 ± 1.5 vs. 45.3 ± 7.1, *P *< 0.0001, and *P *= 0.01, and *P *< 0.0001) (Figure 2).

As for the total circulating EPCs, additional analysis showed no differences in CFU-ECs between patients with versus those without dialysis, and no differences in CFU-ECs between surviving patients and patients with sepsis-related death.

### Correlation analysis

Significant impairment of the EPC system in uremia had already been documented in 2004. Renal patients had significantly fewer EPCs than healthy subjects, and uremic serum markedly inhibited EPC differentiation and functional activity of the cells *in vitro *[18]. Since in our study EPC proliferation was decreased in septic patients, further analysis was performed in order to correlate serum creatinine levels to both the numbers of colonies formed in culture (CFU-ECs assay), and the percentages of peripheral CD133^+^/Flk-1^+ ^cells (circulating EPCs). There was no correlation between the mean serum creatinine levels and the numbers of colonies or the percentages of circulating EPCs in both the 'normal creatinine group' and the 'high creatinine group'.

### Analysis of proangiogenic cytokine levels

Previous studies showed increased serum levels of proangiogenic vascular endothelial growth factor (VEGF) as early as six hours after diagnosing sepsis [25]. In order to assess proangiogenic mediators, we measured serum levels of VEGF, stromal derived factor-1 (SDF-1), angiopoietin-1 (Ang-1), angiopoietin-2 (Ang-2) and fibroblast growth factor (FGF) in all patients and in controls. VEGF and SDF-1 are some of the most potent known activators of EPCs [13,34], while Ang1-/Tie-2 signaling regulates both the maintenance of vascular quiescence and promotion of angiogenesis 1 [35]. Increased angiopoietin-2 expression has been shown in stressed endothelial cells, where it can act as an autocrine protective factor of vascular function [36]. Fibroblast growth factor (FGF) is currently being evaluated as a stimulator of angiogenesis [37]. As for cell analyses, cytokine levels were examined within 12 hours after the diagnosis of sepsis.

**Figure 3:**
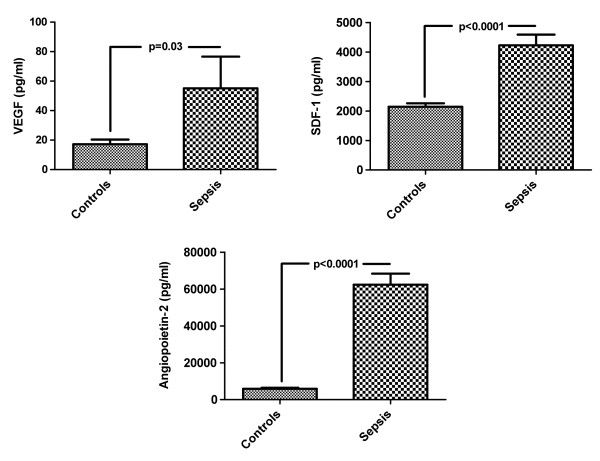
**Serum levels of VEGF, SDF-1, and Ang-2 were dramatically higher in sepsis patients than in healthy controls**. Analysis was performed within 6 to 12 hours after diagnosis of the disease (Results as mean ± SEM).

The serum levels of three mediators, VEGF (55 ± 21 pg/ml vs. 17 ± 3.2 pg/ml, *P *= 0.03), angiopoietin-2 (62,379 ± 6,020 pg/ml vs. 5,892 ± 510 pg/ml, *P *< 0.0001), and SDF-1 (4,223 ± 360 pg/ml vs. 2,143 ± 117 pg/ml, *P *< 0.0001) were significantly higher in patients with sepsis than in healthy controls (Figure 3). Neither serum levels of Ang-1 nor FGF differed between healthy controls and patients with sepsis. The serum levels of VEGF or angiopoietin-2 or SDF-1 did differ between patients within the 'high creatinine group' and the 'normal creatinine group' (data not shown).

## Discussion

The aim of the present study was to analyze the endothelial progenitor cell system in patients suffering from sepsis with acute impairment of renal function. We found a significantly higher mean percentage of circulating EPCs in all sepsis patients that were analyzed. Subgroup analysis showed that patients with a mean serum creatinine concentration above the normal range displayed a strong mobilization of CD133^+^/Flk-1^+ ^cells, whereas, such an increase was absent in sepsis patients with normal mean creatinine levels. In contrast, EPC proliferation was severely affected in sepsis patients. As opposed to previously published results [26], neither total circulating EPCs (CD133^+^/Flk-1^+^) nor EPC proliferation differed between surviving patients and patients with sepsis-related death. Serum levels of proangiogenic VEGF, SDF-1, and angiopoietin-2 were higher in sepsis than in healthy controls.

Our data partly conform with observations made by other investigators. Rafat *et al. *[26] found significantly higher numbers of circulating EPCs (defined as CD133^+^/CD34^+^/Flk-1^+ ^cells) in sepsis patients than in nonseptic intensive care unit patients and in healthy controls. In addition, proangiogenic VEGF was also higher in sepsis, and EPC percentages correlated with patient survival. The authors concluded that EPC enumeration in peripheral blood of septic patients might be of benefit in order to assess the clinical outcome in this condition. Another study, performed by Becchi and colleagues [25], also showed EPC mobilization in sepsis with an even more pronounced increase in severe courses of the disease. Nevertheless, in the latter study EPCs were solely defined by the expression of CD34. This approach is potentially critical since CD34 is substantially expressed on mature endothelial cells and on different types of hematopoietic precursor cells as well [38]. This might explain the significant higher average percentages of EPCs reported in the study [25]. Our analysis did not show different percentages of circulating EPCs between dead and surviving patients. The reason for this discrepancy remains speculative, although it seems possible that the narrower time frame in which blood samples were obtained in our study (12 hours after diagnosing sepsis as opposed to 48 hours in the study by Rafat and colleagues [26]) can account for the different results.

Nevertheless, the most intriguing findings in our study were related to circulating EPCs and EPC proliferation in patients with high mean serum creatinine. Different studies have reported reduced numbers and impaired function of EPCs in chronic renal insufficiency [18,39,40]. These observations mirror the state of generalized endothelial dysfunction in chronic kidney disease (CKD). Mechanisms responsible for EPC suppression, thereby, involve deleterious effects of different substances such as parathyroid hormone (PTH), IL-6, homocysteine, and p-cresol [40]. The patients that were analyzed in our study displayed higher percentages of circulating EPCs, which was in line with previously published data from patients with sepsis [25,26], but opposed to chronic renal failure, acute impairment of renal function did not significantly suppress such an EPC mobilization. Patients within the 'high creatinine group', in contrast, showed an even more pronounced elevation of CD133^+^/Flk-1^+ ^cells. The mobilization of EPCs could be explained as a result of higher mean serum levels of three mediators, all of them known to be involved in stimulating EPC migration (SDF-1 [13], angiopoietin-2 [41], and VEGF [34]). Thus, the influence of acute renal malfunction seems to have a different impact on the EPC system than CKD. A complete lack of any impact can be denied, since especially patients within the 'high creatinine group' showed a significantly stronger EPC mobilization than patients within the 'normal creatinine group'. Pronounced suppression of EPC proliferation might result from a beginning accumulation of endogenous toxins as this is thought to be responsible for EPC suppression in CKD [18]. The higher percentages of circulating EPCs in patients within the 'high creatinine group' are of particular interest since these patients did not display higher average serum levels of the proangiogenic cytokines that were measured. Therefore, acute renal dysfunction possibly activates 'vascular danger signals' in order to activate endogenous repair mechanisms. A number of studies showed that EPCs are potent mediators of renal repair after ischemia [16,23,24]. It has been documented that acute renal ischemia, since it is the most frequent cause of acute renal failure in the intensive care unit, dramatically mobilizes EPCs from their respective niches. This mobilization occurs as early as three hours after hypoperfusion [16]. A very potent endogenous mediator of EPCs is uric acid which is rapidly released into systemic circulation after reperfusion has been initiated [23]. Uric acid-mediated EPC mobilization results from degranulation of Weibel-Palade bodies and this event requires the presence of toll-like receptor 4 (TLR 4) [41]. Since TLR 4 acts as the receptor that signals LPS bioactivity in sepsis [42], the TLR4/uric acid/Weibel-Palade axis might work as the proposed 'vascular danger signals' that agonizes EPCs in the bone marrow to migrate into the circulation. Patients within the 'high creatinine group' showed significantly higher serum levels of uric acid, which is in line with the proposed hypothesis of uric acid mediated EPC mobilization in sepsis-associated acute renal dysfunction. Nevertheless, the possible role of uric acid as endogenous stimulator of EPC mobilization in the setting of sepsis can only be speculated at the moment and further analysis will have to be performed in order to further confirm this theory.

In summary, we present the first data on EPC mobilization and proliferation in sepsis with acute impairment of renal function. Acute renal dysfunction, via increasing serum concentrations of endogenous toxins, augments sepsis-associated EPC mobilization and worsens suppression of EPC proliferation. The molecular mechanisms responsible for increased cell mobilization involve increased production and release of proangiogenic substancies. In addition, regarding the literature on the mechanisms of post-ischemic EPC mobilization and regarding systemic concentrations of uric acid a proposed 'vascular danger cascade' might involve release of uric acid and actions of TLR 4. This possible relationship has to be analyzed in further studies.

## Conclusions

In conclusion, sepsis is associated with significant impairment of the endothelial progenitor cell system. This is reflected by increased EPC numbers, increased concentrations of proangiogenic mediators, and reduced proliferative capacity of the cells, respectively. While these events occur independently from the frequency of dialysis and from patient survival, increased serum levels of uric acid could potentially play a role in the stimulation of EPC mobilization in sepsis patients with higher average creatinine levels.

## Key messages

• The endothelial progenitor cell system is severely affected in sepsis.

• Sepsis patients with higher mean serum creatinine levels, due to acute kidney injury, show an even more pronounced mobilization of EPCs.

• Alterations of the EPC system in sepsis occur independently from the frequency of dialysis and independently from patient survival.

## Abbreviations

AKIN: acute kidney injury network; ARD: acute renal dysfunction; CFU: colony forming unit; DiI-ac-LDL: acetylated low density lipoproteins, labeled with 1,1\'-dioctadecyl - 3,3,3\',3\'-tetramethyl-indocarbocyanine perchlorate; EPCs: endothelial progenitor cells; ESRD: end stage renal disease; FGF: fibroblast growth factor; Flk-1: fetal liver kinase-1; ICU: intensive care unit; MNCs: mononuclear cells; SAPS II: simplified acute physiology score II; SDF-1: stromal derived factor-1; SIRS: systemic inflammatory response syndrome; UE lectin: ulex europaeus lectin; VEGF: vascular endothelial growth factor.

## Competing interests

The authors declare that they have no competing interests.

## Authors' contributions

SP designed the study, collected blood samples from the patients, analyzed the data and wrote parts of the manuscript. DP participated in the design of the study, included and followed patients, assisted in analysis of the data and wrote parts of the manuscript. JT collected blood samples and documented clinical data from the patients. PK assisted in writing the manuscript. JW performed microscopic analysis and counted cell colonies. MK helped in analyzing the data. EH performed cell culture experiments, cytometric analysis and ELISA studies. GAM initiated the study, participated in the data analysis and wrote parts of the manuscript.
